# Long‐term fasting induces a remodelling of fatty acid composition in erythrocyte membranes

**DOI:** 10.1111/eci.14382

**Published:** 2025-01-13

**Authors:** Katharina Gewecke, Franziska Grundler, Massimiliano Ruscica, Clemens von Schacky, Robin Mesnage, Françoise Wilhelmi de Toledo

**Affiliations:** ^1^ NÀDARRA GmbH Hamburg Germany; ^2^ Buchinger Wilhelmi Clinic Überlingen Germany; ^3^ Department of Pharmacological and Biomolecular Sciences “Rodolfo Paoletti” Università Degli Studi di Milano Milan Italy; ^4^ Department of Cardio‐Thoracic‐Vascular Diseases Foundation IRCCS Ca’ Granda Ospedale Maggiore Policlinico Milan Italy; ^5^ Omegametrix Martinsried Germany; ^6^ Department of Nutritional Sciences, Faculty of Life Sciences and Medicine School of Life Course Sciences, King's College London London UK

**Keywords:** adipose tissue, erythrocyte, fasting, fatty acids, long‐term fasting, Omega‐3 index

## Abstract

**Introduction:**

Long‐term fasting (LF) activates an adaptative response to switch metabolic fuels from food glucose to lipids stored in adipose tissues. The increase in free fatty acid (FFA) oxidation during fasting triggers health benefits. We questioned if the changes in lipid metabolism during LF could affect lipids in cell membranes in humans. We thus analysed the FA composition in erythrocyte membranes (EM) during 12.6 ± 3.5 days of LF and 1 month after food reintroduction.

**Methods:**

A total of 98 subjects out of three single‐arm interventional studies underwent a medical supervised long‐term fasting (12.6 ± 3.5 days) programme. The distribution pattern of 26 FA as well as the HS‐Omega‐3 Index were assessed in the EM using gas chromatography.

**Results:**

Eighteen of 26 FA showed significant changes. Within the group of saturated FA, myristic (14:0) and stearic acid (18:0) decreased while palmitic (16:0) and arachid acid (20:0) increased. While most monounsaturated FA increased, trans fatty acids decreased or remained unchanged. Within the polyunsaturated FA, arachidonic (20:4n6) and docosahexaenoic (22:6n3) acid increased, while linoleic (18:2n6), alpha‐linolenic (18:3n3) and eicosapentaenoic acid (20:5n3) decreased. Consequently, the HS‐Omega‐3 Index increased. 11 out of the 18 FA with significant changes returned to baseline levels 1 month afterwards. Levels of linoleic and alpha‐linolenic acid increased over baseline levels.

**Conclusions:**

Long‐term fasting triggers changes in the FA composition of EM.

## Graphical abstract

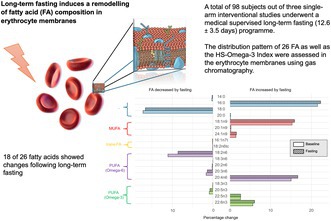

## 1 INTRODUCTION

Fasting represents an adaptive response that allows both animals and humans to manage periods of food scarcity by mobilizing stored energy sources, mainly fat from the adipose tissues. The health‐promoting effects of long‐term fasting (LF), lasting from 4 days up to several weeks, are increasingly documented.^1^, ^2^ This includes studies showing that LF improves lipid profile and lipoproteins' functionality to reduce cardiovascular risk factors,^3^ metabolic dysfunction‐associated fatty liver disease,^4^ hypertension,^5^ chronic inflammation, oxidative stress^6^ and gut microbiota profiles.^7^

Fatty acids (FA) play different roles in the human body: as fuel, structural components and signalling molecules. Previous studies, including our own,^8^ have documented the use of fat to meet energy demand during LF. However, to our knowledge, structural fats—such as those found in the white and grey matter of the brain, in cell membranes providing fluidity and permeability, and the myelin sheath insulating nerve fibres—have not been specifically targeted in human fasting research. EM fluidity is even proposed as a biomarker of residual cardiovascular risk in type 2 diabetes.^9^ In the present study, we have focused on examining the fat composition of erythrocyte membranes (EM). The turnover of fatty acids in erythrocyte membranes of fed subjects is relatively slow, typically occurring over the lifespan of the erythrocyte, which is approximately 120 days.^10^ This slow turnover rate reflects the stable nature of their membrane composition.

Considering fat as fuel, it is documented that after 12–36 h of fasting, depending on the energy content of the last meal and the energy expenditure, FA and derived ketone bodies replace glucose as primary fuel. This metabolic switch occurs when glycogen stores are depleted, and fat tissue lipolysis generates FA and glycerol.^11^ Free FA (FFA) are released in the blood stream and transported to all organs as fuel and to the liver, where hepatocytes break part of them down into ketone bodies.^12^ The metabolic switch leading to ketosis during fasting contributes to some of its health benefits.^8^

We questioned if the changes in lipid metabolism during LF could affect lipids in cell membranes in humans like it was reported previously in animals.^13^ When the body initiates lipolysis to utilize stored fats for energy, lipid membranes might also serve as a potential energy source or provider of specific FA particularly the essential ones.^14^ It could be postulated that the EM may even incorporate FA provided by fat tissues to maintain membrane integrity. Furthermore, we do not know if prolonged fasting changes the turnover rate of erythrocytes and their membranes. Several studies showed changes in bone marrow fat and erythropoietic tissues following LF.^15^

FA profiles in EM can be good markers of health status. For instance, this applies to the group of essential FA such as omega‐3 and omega‐6. Levels of eicosapentaenoic (EPA) and docosahexaenoic acid (DHA) are among others associated with cardiovascular prevention^16^, ^17^ while it is not clear whether this is the case for levels of alpha‐linolenic acid (ALA).^18^, ^19^, ^20^, ^21^ Unfortunately, with modern nutrition patterns levels of the long‐chain omega‐3 fatty acids EPA and DHA are suboptimal in many countries worldwide (^22^). A standardized analysis of 26 FA was established, including the HS‐Omega‐3 Index describing the sum of long‐chain omega‐3 FA in EM. The levels of the HS‐Omega‐3 Index were among others associated with cardiovascular risk^23^ and brain health^24^ as well as pregnancy outcomes.^25^

We analysed levels of 26 FA in EM from 98 subjects undergoing LF according to the Buchinger Wilhelmi fasting protocol.^26^ We also followed patients 1 month after fasting to understand the persistence of possible fasting‐induced changes in FA levels.

## 2 METHODS

### 2.1 Study design

**FIGURE 1:**
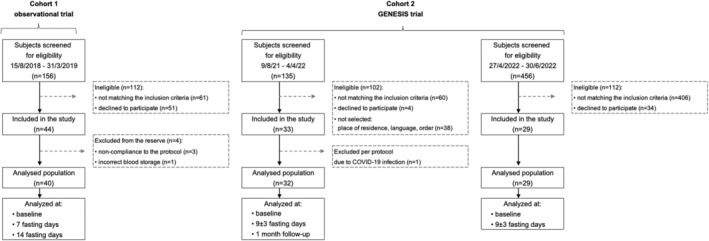
Flow chart of the selection procedure. A total of 98 subjects underwent a medical supervised long‐term fasting program. The distribution pattern of 26 fatty acids was measured in the erythrocyte of these patients.

We included a total of 98 subjects from two study cohorts. Figure 1 illustrates the composition of the participant pool, which consisted of 40 subjects from an observational trial^27^ and 58 subjects from the GENESIS trial.^28^ Within the GENESIS cohort, there were 32 subjects who underwent a comprehensive analysis plan including magnetic resonance imaging (MRI) measurements and on‐site follow‐ups. Additionally, 26 subjects were recruited from the in‐patient population of the Buchinger Wilhelmi Clinic (BWC) in Überlingen, who underwent less intensive monitoring without MRI and follow‐up. The BWC is a specialized centre for long‐term therapeutic fasting under medical supervision. Both studies were conducted in adherence to the principles outlined in the Declaration of Helsinki, and all participants provided written informed consent.

The observational prospective study received approval from both the medical council of Baden‐Wurttemberg and the Ethics Committee of the Charite‐University Medical Center, Berlin (application number: EA4/054/15) on 5 May 2015. The study protocol was registered on 3 June 2016 in the German Clinical Trials Register (DRKS‐ID: DRKS00010111). Subjects were recruited at the BWC in Überlingen, Germany, between August 2018 and March 2019.

The GENESIS study, on the other hand, is a prospective, monocentric, single‐arm interventional study with a two‐stage design. The study protocol F‐2021‐075 and its amendment were approved by the medical council of Baden‐Wurttemberg on 7 October 2021. It was registered on clinicalTrials.gov (NCT05031598) before the recruitment phase, which took place between August 2021 and June 2022.

### 2.2 Participants

In the observational study, a total of 40 subjects (50% women) between 30 and 65 years participated, as described in.^27^ These subjects were selected from the in‐patient pool of the BWC based on their completion of the Buchinger Wilhelmi fasting program, enduring a minimum of 14 days and up to 26 days of fasting. The intake of lipid‐lowering drugs, diabetes drugs, and language barriers or the prevalence of contraindications of fasting as defined in peer‐reviewed guidelines^26^ led to exclusion. Blood samples were collected at baseline, after 7 and 14 fasting days.

In another cohort (GENESIS cohort) undergoing the same Buchinger Wilhelmi fasting programme, both women and men aged between 20 and 75 years were included. A total of 58 (50% women) subjects participated. Exclusion criteria encompassed the use of medication for cardiovascular diseases, as well as medications affecting lipid and glucose metabolism. A detailed list of inclusion and exclusion criteria can be found in.^28^ The subjects in the GENESIS cohort underwent a fasting period of 9 ± 3 days. Blood samples for the measurement of the FA in the erythrocyte membrane were collected at baseline and at the end of the fasting period. Among the 32 subjects (50% women) who participated in MRI measurements, a follow‐up analysis was performed after 1 month. Figure 1 shows the selection procedure for both trials.

### 2.3 Fasting protocol

All subjects underwent a medically supervised LF program.^26^ Prior to the start of the fasting, subjects followed a plant‐based, organic, calorie‐restricted diet for 1 day (600 kcal/day). Throughout the fasting period, energy intake was limited to 200–250 kcal/day, which consisted of .25 L freshly squeezed, organic fruit juice at noon, .25 L vegetable soup in the evening and 20 g honey. Subjects were advised to consume a minimum of 2 L of water or non‐caloric, caffeine‐free herbal teas. Every other fasting day, the colon was emptied using an enema or a laxative. On the final day of fasting, a gradual reintroduction of plant‐based, organic food took place over three to four consecutive days, with caloric intake ranging from 800 to 1600 kcal/day. The fasting intervention was closely monitored by medical doctors and nurses. The fasting program is a multidisciplinary program including physical exercise. Further details can be found in previously published articles.^28^

### 2.4 Laboratory examinations

Blood samples were drawn by trained medical‐technical assistants in the morning between 07:30 and 09:30 h. The baseline collection was conducted on the transition day or the first day of fasting in the beginning of the stay at BWC. For the cohort of the observational study, the second blood sample was collected 7 and the third blood sample 14 days after the baseline blood draw. For the GENESIS cohort the second blood sample was drawn 9 ± 3 days after baseline depending on the individual length of the fast. For the participants who participated in MRI measurements a third blood collection took place 1 month after baseline. A centrifugation of serum tubes (S‐Monovette, 9 mL Z‐Gel) was conducted at 3290 ***g*** (5000 rpm) for 10 min at room temperature. The fresh blood samples were used to measure among others, total cholesterol (TC), high‐density cholesterol (HDL‐C) and low‐density lipoprotein cholesterol (LDL‐C). The samples were then stored at −70°C in aliquots for further analysis of lipid profiles, density gradient ultracentrifugation and NMR spectroscopy within 12 months after the blood collection.

### 2.5 Fatty acid analysis

The methodology of how the HS‐Omega‐3 Index methodology has been previously described.^29^ Samples were anonymized before fatty acid analysis. Via acid transesterification fatty acid methyl esters were generated from erythrocytes. Methyl esters were then analysed by gas chromatography using hydrogen as carrier gas with a GC2010 gas chromatograph (Shimadzu, Duisburg, Germany) equipped with an SP2560, 100‐m column (Supelco, Bellefonte, PA). To identify the fatty acids, the results were compared to a standard mixture of FA characteristic of erythrocytes. After a response factor correction, the Omega‐3 Index is given as the sum of EPA and DHA expressed as the share (in %) of the total identified FA.^29^ The variation coefficient for the Omega‐3 Index was 5%. DIN ISO 15189 was applied for quality control of the analyses.

### 2.6 Statistical analysis

The statistical analysis of the data was performed using R software for statistical computing version 4.2.2 (2022‐10‐31). Given the longitudinal design of the study with repeated measurements, linear mixed models using the subject unique ID as a random effect and the timepoint as a fixed effect were performed using the R package lmerTest. The *p*‐values from linear mixed models in the longitudinal data analysis were adjusted with a post‐hoc Tukey test. The statistical models were adjusted for potential confounders such as age, gender and body mass index (BMI). If the interactions were not statistically significant and contributing to the model, the interaction term was dropped to make the model more parsimonious.

## 3 RESULTS

### 3.1 Subject characteristics

Screenings for study eligibility were performed between August 2018 and March 2019 for the observational study and from August 2021 to June 2022 for the GENESIS study (Figure 1). A total of 747 subjects were screened for eligibility. Of these, 106 were enrolled. One subject was excluded from the analysis due to incorrect blood processing, three due to minor noncompliance, another one due to SARS‐CoV‐2 infection and three due to drop‐out. Data were available for 98 subjects who fasted for an average of 13 fasting days (Table 1). Subject characteristics of the three groups were mostly comparable (Table 1).

**TABLE 1:** Characteristics of the subjects.

	Observational study	GENESIS MRI	GENESIS	Total	*p* Value
Fasting duration days	14	11	9 ± 3	12.6 ± 3.5	
*n*	40	32	26	98	
Men (%)	20 (50)	16 (50)	13 (50)	49 (50)	
Women (%)	20 (50)	16 (50)	13 (50)	49 (50)	
Age years	50.7 ± 9.3	47.9 ± 12.1	53.9 ± 12.2	50.6 ± 11.4	.13
Weight kg	89.6 ± 19.8	79.4 ± 12.8	80.6 ± 11.4	83.9 ± 16.6	.02*
BMI kg/m^2^	29.8 ± 5.4	26.2 ± 3.8	27 ± 3.4	27.9 ± 4.8	.003**
Total cholesterol mmol/L	5.52 ± 1.4	5.54 ± .95	5.66 ± 1.0	5.57 ± 1.29	.9
LDL cholesterol mmol/L	3.21 ± 1.1	3.68 ± .95	3.76 ± 1.35	3.51 ± 1.17	.11
HDL cholesterol mmol/L	1.35 ± .4	1.68 ± .41	1.56 ± .5	1.52 ± .45	.007**

*Note*: Values are indicated as mean ± SD. The baseline characteristics of the cohorts were compared using an ANOVA test (*p < .05; **p < .01).

### 3.2 Changes of anthropometric, lipid and erythrocyte biomarkers

In all groups, fasting resulted in a significant weight loss. On average, participants lost −6.08 kg during the fasting period, and 1.57 kg was regained after a month (Table S1). Triglycerides, HDL‐C and LDL‐C were reduced. Haematocrite, red cell distribution width (RDW), and mean corpuscular volume (MCV) decreased, while mean corpuscular haemoglobin concentration (MCHC) increased during fasting. The changes in MCV and MCHC persisted 1 month afterwards.

### 3.3 Changes in the FA pattern during the fasting

**FIGURE 2:**
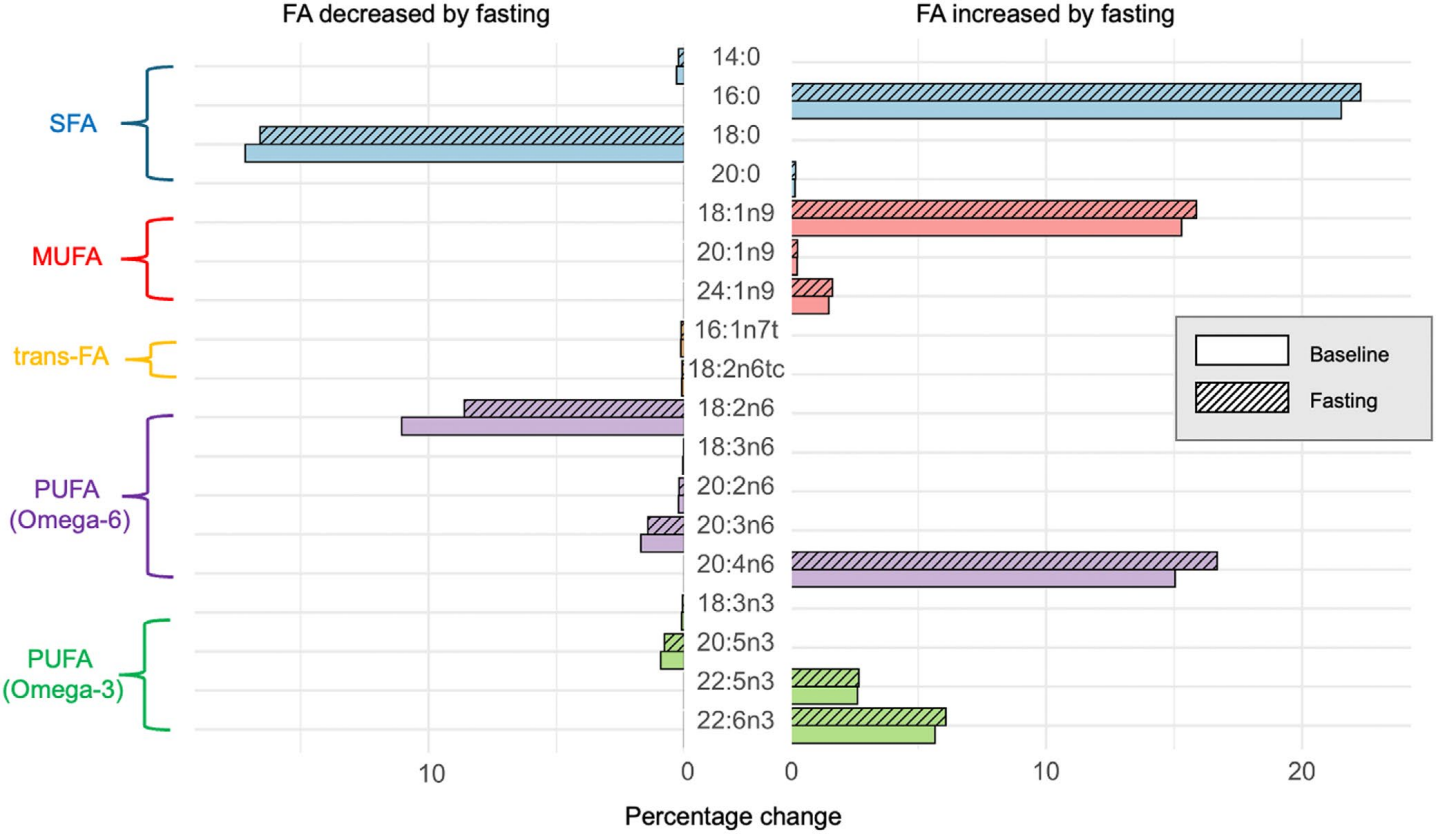
Erythrocyte fatty acids decreasing or increasing significantly during long‐term fasting (all cohorts, *n* = 98).

The distribution pattern of 26 FA in the EM and the HS‐Omega‐3 Index were analysed (Table 2). For ten of the FA, a significant decrease in the EM was observed, while eight FA significantly increased in the EM (Figure 2). The HS‐Omega‐3 Index slightly, but significantly increased during LF (+.26). There was no interaction between the age or sex of the participants with the effects of fasting on the different FA.

**TABLE 2:** Changes in FA composition of EM during long‐term fasting.

		Baseline (%)	Fasting (Δ)	Follow‐up (Δ)
14:0	Myristic acid	.31	−.08***	+.03
16:0	Palmitic acid (PA)	21.53	+.76***	+.08
18:0	Stearic acid	17.16	−.58***	−.16
20:0	Arachid acid	.19	+.01***	−.01
22:0	Behenic acid	.49	+.01	−.08***
24:0	Lignoceric acid	1.22	+.02	−.06
16:1n7	Palmitoleic acid	.27	+.02	−.03
18:1n9	Oleic acid (OA)	15.29	+.58***	+.37
20:1n9	Gadoleic acid	.27	+.03***	.02**
24:1n9	Nervonic acid	1.51	+.14***	−.20**
16:1n7t	Trans‐Palmitoleic acid	.14	−.01*	−.01**
18:1 t	Trans‐Oleic acid	.75	−.05	−.04
18:2n6tt	Trans‐Linoleic acid (tt)	.01	.0	.0
18:2n6ct	Trans‐Linoleic acid (ct)	.02	.0	.0
18:2n6tc	Trans‐Linoleic acid (tc)	.10	−.01**	.0
18:2n6	Linoleic acid (LA)	11.05	−2.45***	+.97***
18:3n6	Gamma‐linolenic acid	.06	−.03***	−.01*
20:2n6	Eicosadienoic acid	.23	−.01***	.0
20:3n6	Dihomo‐gamma‐linolenic acid	1.70	−.27***	−.12***
20:4n6	Arachidonic acid (AA)	15.04	+1.64***	−.29
22:4n6	Docosatetraenoic acid	2.72	+.04	−.02
22:5n6	Osbond acid	.58	−.01	−.03
18:3n3	Alpha‐Linolenic acid (ALA)	.11	−.03***	.06***
20:5n3	Eicosapentaenoic acid (EPA)	.93	−.16***	+.01
22:5n3	Docosapentaenoic acid	2.63	+.05*	−.03
22:6n3	Docosahexaenoic acid (DHA)	5.66	+.42***	−.18
HS‐Omega‐3 Index		6.59	+.26***	−.17

*Note*: Baseline represents the initial measurement of FA relative abundance (all cohorts, *n* = 98). Fasting indicates the change (Δ) in FA relative abundance during the fasting period (all cohorts, *n* = 98). Follow‐up shows the change (Δ) in FA relative abundance at the follow‐up timepoint in comparison to baseline (GENESIS MRI cohort, *n* = 32). The statistical significance of changes is indicated as follows: *p < .05, **p < .01, and ***p < .001.

The directionality of the changes compared to baseline is indicated by red (decrease) or green (increase) shading.

Considering the saturated fatty acids (SFA), myristic (14:0) and stearic acid (18:0) significantly decreased, while palmitic acid (PA, 16:0) and arachid acid (20:0) significantly increased. The long‐chained SFA behenic (22:0) and lignoceric acid (24:0) did not show a significant change. The shortest monounsaturated fatty acid (MUFA) palmitoleic acid (16:1n7) did not significantly change during the fasting, while all other MUFA—oleic (OA, 18:1n9), gadoleic (20:1n9) and nervonic acid (24:1n9)—significantly increased. Of the trans‐FA, none is showing a significant increase, while trans‐palmitoleic (16:1n7t) and trans‐linolenic acid (18:2n6tc) decreased and trans‐oleic (18:1 t) and trans‐linoleic acid (18:2n6tt, 18:2n6ct) did not change significantly. Of the omega‐6 polyunsaturated fatty acid (PUFA) family, the comparatively shorter FA linoleic (LA, 18:2n6), gamma‐linolenic (18:3n6), eicosadienoic (20:2n6) as well as dihomo‐gamma‐linolenic acid (20:3n6) decreased significantly. However, arachidonic acid (AA, 20:4n6) increased and the very long‐chained docosatetraenoic acid (22:4n6) and 22:5n6 did not change significantly. The omega‐3 PUFA family showed the same pattern: The shorter FA ALA (18:3n3) and EPA (20:5n3) decreased, while the longer ones docosapentaenoic (22:5n3) and DHA (22:6n3) increased significantly (Table 2).

Within the group of FA that decreased, stearic and LA constitute the largest proportion within the EM (17.16% and 11.05%, respectively, at baseline), and they also showed the most prominent decreases with LA going down by −2.45 and stearic acid by −.58 (Table 2). Of the FA increasing, PA, OA and AA had the significantly highest proportion within the EM (21.53%, 15.29% and 15.04%, respectively, compared to baseline). AA increased by +1.64, PA by +.76 and OA by +.58 (Figure 2). FA that exhibited minimal changes shared a common characteristic: their levels in the EM were relatively low, with docosatetraenoic acid comprising the largest portion at 2.72% initially. Their changes during the fasting period were minimal, typically around ±.04 or even less.

### 3.4 Effects after 7 and 14 days of fasting

For the cohort of the observational study (*n* = 40), measurements were performed after 7 and 14 days of fasting (Figure S1). This provided us with an opportunity of understanding the kinetic of these changes. Looking at the FA with the most prominent changes it shows that the more pronounced effect is observed within 7 days of fasting. LA decreased by −1.86 in the first 7 days and by −.89 from day seven to fourteen. Likewise, AA and DHA increased by 1.27 and .41 in the first half of the fasting and by another .50 and .12, respectively, in the second half. This suggested that changes during fasting might decrease exponentially.

### 3.5 Sustainability of the fasting effects

For 32 subjects of the GENESIS MRI cohort, follow‐up data was available from 1 month after the fasting. A general observation is that FA that decreased during the fasting increased afterwards. In the group of SFA, PA and stearic acid showed the most prominent changes in comparison to baseline (−.69 and +.41, respectively). In the group of MUFA and trans‐FA most of the FA did not significantly differ from baseline. Within the omega‐6 and omega‐3 families there was a trend that the shorter FA end up increased while the longer forms decreased. LA and AA showed the most marked change with LA rising by +3.42 and AA decreasing by −1.92. While EPA did not change significantly compared to baseline, DHA decreased by −.6 and thus the HS‐Omega‐3 Index also returns to a lower level.

## 4 DISCUSSION

Our study provides observations of the remodelling of FA profiles in EM during long‐term fasting in humans. In fed subjects, the composition of a single erythrocyte is known to be stable during the lifespan around 120 days. Its composition is dependent on exogenous dietary intake, particularly for the essential fatty acids, pool size and utilization. As a consequence, it was recently suggested to be used as an appropriate biomarker to investigate correlations between FA metabolism and diseases.^30^, ^31^, ^32^

In the present study, we found EM FA composition at baseline corresponding to usual values (Table 2). During fasting, we observed a substantial change in the FA composition of EM already after 12 ± 3 days of fasting. Of the 26 FA which have been measured, 10 decreased, eight increased and the remaining eight were unchanged. Six of the 10 FA which decreased significantly during LF belong to the two families of essential and semi‐essential omega‐6 and omega‐3 FA. The shortest FA within these families, LA and ALA are defined as essential since their first double bond from the methyl ending cannot be synthesized by the human body. The omega‐3 and omega‐6 FA with longer carbon chain length are considered semi essential: they can be obtained via several steps of elongation, and desaturation of LA and ALA in cell mitochondria. In our study, LA which is a major component of EM substantially decreased as well as ALA, gamma‐linolenic, eicosadienoic, dihomo‐gamma‐linoleic and EPA (Table 2).

Considering the PUFA that increase, we noted a rise in AA and DHA. It is conceivable that rates of elongation and desaturation of long‐chain semi‐essential FA are increased during LF and/or that they are actively retained from being oxidized. They serve as building blocks for tissue hormones (prostaglandins) and lipid mediators,^33^ rendering them essential for the functioning of the brain and for cardiovascular health.^23^, ^24^, ^34^ Another study observed a shift to longer and more highly unsaturated fatty acids in plasma during LF^35^ which would support the hypothesis that these FA are actively held back from oxidation and could enter the erythrocytes. However, this hypothesis does not apply to EPA which is known to reduce the risk of ischemic events,^36^ although the efficacy of the dosage used in this aforementioned study can be questioned. A similar mechanism may apply to nervonic acid that increase like AA and DHA. It is associated with brain and nerve function,^37^ which could be a reason why our metabolism has evolved to retain it during fasting.

Two trans‐FA out of 5 are decreasing. This includes trans‐linoleic and trans‐palmitoleic acid. These trans‐FA are unhealthy and can be found in industrial products. Since elevated levels of industrial trans‐FA are associated with cardiovascular disease^38^ a decrease in their levels could be beneficial. Further studies in patients who consume large amounts of ultra‐processed food could reveal more benefits at this level.

During fasting, the different adipose tissues become the main providers of FFA. To our knowledge, the changes in composition of FA of the different adipose tissues in humans is scarcely documented during fasting, whereas this is more described in other vertebrates.^13^ Although we did not perform adipose tissue biopsies, the composition of FA stored in the adipose tissues is predominantly determined by de novo lipogenesis in the liver with palmitate as a common precursor.^39^ Consequently, adipose tissues exhibit a substantial proportion of palmitate‐derived FA, wherein OA constitutes the largest fraction at 43.5%, followed by PA at 21.5% and LA at 13.9%.^30^ We hypothesized that the composition of the FA in adipose tissue might influence the changes in EM during LF. However, in our study, PA levels increase while LA levels decrease during fasting.

An additional factor determining changes in FA levels in EM is their preferential utilization in the beta oxidation. Specifically, FA with more double bonds and those with shorter chains are preferentially mobilized.^40^ While this pattern is only partially reflected in our results—with contrasting effects on different SFA—a combination of effects from both preferential utilization and alterations in the supply source could better explain these changes. For instance, a substantial proportion of PA might undergo oxidation for energy production, even as its proportion in the erythrocyte membrane increases owing to its supply from adipocytes, where it is the most abundant FA (43.5%).

It's crucial to note that energy metabolism undergoes fundamental shifts during fasting, and norms established for individuals in a fed state may not be directly applicable. Sparing mechanisms may come into play, allowing the organism to conserve essential FA from being directed towards oxidation.

PA is associated with metabolic and cardiovascular diseases.^41^, ^42^ Previous studies have indicated that prolonged caloric restriction and weight loss over an extended period (8 weeks to 6 months) among individuals with significant visceral fat, correlated with reductions of PA in both erythrocytes and plasma.^43^, ^44^ While our study observed an elevation in PA during LF, this increase was only temporary.

The HS‐Omega‐3 Index has an optimum at 8%–11% and describes the sum of the long‐chain omega‐3 FA EPA and DHA also described as marine fatty acids, since they are found in sea food, fatty fish and algae. Both EPA and DHA can confer health benefits but they have different biological properties.^45^, ^46^ Epidemiological and interventional studies have consistently demonstrated that the Omega‐3 Index is predominantly influenced by the regular intake of the fatty acids it encompasses.^16^ Given this scientific foundation, one might expect a decline in the Omega‐3 Index during LF, as there is an absence of exogenous provision of the long‐chain omega‐3 PUFA. Surprisingly, in our study cohort, the Omega‐3 Index exhibited an increase. While the mechanistic underpinning this phenomenon is to be elucidated, the present observation suggests that in the course of evolution, mechanisms to safeguard these fatty acids have emerged. No human being was found with an HS‐Omega‐3 Index <2%.^47^ This is also true for vegan individuals not ingesting EPA and DHA.^47^ This implies that elongation enzymes are activated, or some mechanisms of retention are at play. The metabolic shift induced by LF appears to be beneficial to maintain homeostasis, even in the absence of exogenous EPA and DHA supply.

The typical lifespan of normal human red blood cells in the bloodstream is approximately 120 days.^48^ It is thus surprising to see pronounced changes in the present fasting intervention. However, it did not inform on a possible acceleration of the turnover which could be hypothesized to explain how the FA patterns of the EM could change so rapidly. A link to inflammation can be hypothesized. On one hand, fasting has known anti‐inflammatory effects.^1^ On the other hand, FA patterns of the EM and red cell distribution can be influenced by the inflammatory status.^49^, ^50^, ^51^

Supplementation in dietary FA, of EPA and DHA, showed an elevation in the EM after 4 to 6 months followed by a steady state.^52^ However, intervention studies also showed that changes in FA levels are already significant after 14 days depending on the consumed dose of EPA or DHA^53^, ^54^ which demonstrates that the remodelling is always ongoing. An intervention giving different doses of EPA observed, that already at the smallest dose of 440 mg EPA per day the Omega‐3 Index increased by +.29 within the first 2 weeks^54^ which corresponds to the changes we observed. Since the number of erythrocytes isn't changing during our intervention and their volume is changing statistically significant, but probably clinically irrelevant (Table S1), an active process of replacing of FA in the membrane seems to take place.

Ketosis during fasting may have an erythropoietic effect. In mice, ketosis led to the inhibition of class I histone deacetylases,^55^ key regulators in the modulation of erythropoiesis.^56^ However, it is still unclear whether fasting could lead to an acceleration or a deceleration of the turnover of erythrocytes. A study focusing on mice fasted for 48 h revealed a delayed process of erythroid differentiation and maturation.^57^ But in a recent study posted as a preprint, a short‐term fasting intervention was suggested to trigger rejuvenation of erythropoiesis by regulating autophagy‐associated pathways.^58^ Altogether, whether the changes observed in this study reflect an acceleration of the red blood cells turnover remains to be explored.

The follow‐up data suggests that the changes of the FA pattern caused by fasting is reversible. The change to a more plant‐based diet rich in nuts, seeds and vegetarian/vegan products containing plant oils with high contents of LA and ALA could explain the elevation in LA levels 1 month post fasting.^59^

The results of our study need to be interpreted in the context of some limitations. First, we cannot fully understand the kinetic of the observed changes, because FA profile was not studied either in the first days of fasting, or in the first days of the food reintroduction. Second, there is no control group to rule out the influence of other factors than fasting itself. The single‐arm design thus limits the ability to understand potential influences (e.g., baseline diet, physical activity). Third, our study was not well‐powered to detect any influence of sex and age on the effects of fasting. Fourth, a longer follow‐up could offer better insights into the persistence or further evolution of lipid changes. Finally, while erythrocyte membranes are a valuable proxy for systemic lipid metabolism, the evaluation of circulating lipids could provide a more comprehensive view.

What surprised us most were the fast and significant changes that we observed. Explanatory approaches can be found in the fasting metabolism: Instead of exogenous supply the body uses its own resources, with fat depots depicting the most effective and largest storage capacity for energy. During fasting, the adipose tissue is the main pool for energy supply in the form of free FA. Hence, the FA pattern of the erythrocytes might be influenced by the pattern of FA in adipocytes as well as a preferential release and oxidation of FA. Moreover, sparing mechanisms might enable our organism to save important FA from going into oxidation. Most likely, further mechanisms haven't been revealed yet.

## 5 CONCLUSION

In conclusion, our investigation into the impact of LF on EM FA levels shows a nuanced response, revealing both decrements and increments in specific FA. The observed elevation in DHA contributes to an improved HS‐Omega‐3 Index, suggesting potential health benefits; however, this effect is not maintained during follow‐up. These findings provide valuable insights into the intricate metabolic adaptations induced by long‐term fasting, paving the way for targeted therapeutic strategies to enhance cardiovascular and metabolic health.

## AUTHOR CONTRIBUTIONS

Conceptualization: FWT and FG; data curation and analysis: RM, KG and FG; writing—original draft: KG, RM, FG; writing—review and editing: FWT, CvS, KG, RM and FG.

## FUNDING INFORMATION

The study was financed by Buchinger Wilhelmi Development & Holding GmbH.

## CONFLICT OF INTEREST STATEMENT

Authors FG, RM and FWT are employees of the Buchinger Wilhelmi Development and Holding GmbH. CvS was employed by Omegametrix. KG has been employed by NORSAN GmbH.

## Supporting information


Appendix S1.

## Data Availability

The data that support the findings of this study are available from the corresponding author upon reasonable request.
