# Supplementary material for: Long‐term fasting induces a remodelling of fatty acid composition in erythrocyte membranes
Source: Eur J Clin Invest. 2025 Jan 13;55(5):e14382. doi: 10.1111/eci.14382 (PMC12011679; doi:10.1111/eci.14382)
Supplement: Supplementary file 1 — Appendix S1. [file ECI-55-e14382-s001.docx]

# Supplementary material

Table S1: Changes of blood markers during long-term fasting and one month afterwards.

Values represent the difference of the mean to the baseline mean of the associated cohort (* p<0.05; ** p<0.01; *** p<0.001).

|  | **baseline** | **fasting** | **follow-up** |
| --- | --- | --- | --- |
| **cohort** | total | total | GENESIS MRI |
| **n** | 98 | 98 | 32 |
| **Weight** kg | 83.9 ± 16.6 | -6.08*** | -4.51*** |
| **BMI** kg/m² | 27.9 ± 4.8 | -2.01*** | -1.49*** |
| **Total cholesterol** mmol/L | 5.57 ± 1.29 | -0.95*** | -0.21 |
| **LDL cholesterol** mmol/L | 3.51 ± 1.17 | -0.72*** | -0.20 |
| **HDL cholesterol** mmol/L | 1.52 ± 0.45 | -0.23*** | 0.11* |
| **Erythrocytes** | 4.72 ± 0.51 | 0.01 | 0.02 |
| **Hematocrit** | 41.25 ± 3.71 | -0.61** | 0.31 |
| **RDW** | 12.95 ± 0.82 | -0.27*** | NA |
| **MCV** | 87.72 ± 3.63 | -1.42*** | -1.41*** |
| **MCH** | 30.16 ± 1.38 | 0.02 | -0.05 |
| **MCHC** | 34.39 ± 0.90 | 0.58*** | -0.59*** |
| **Hemoglobin** | 14.71 ± 1.37 | 0.09 | 0.14 |

**
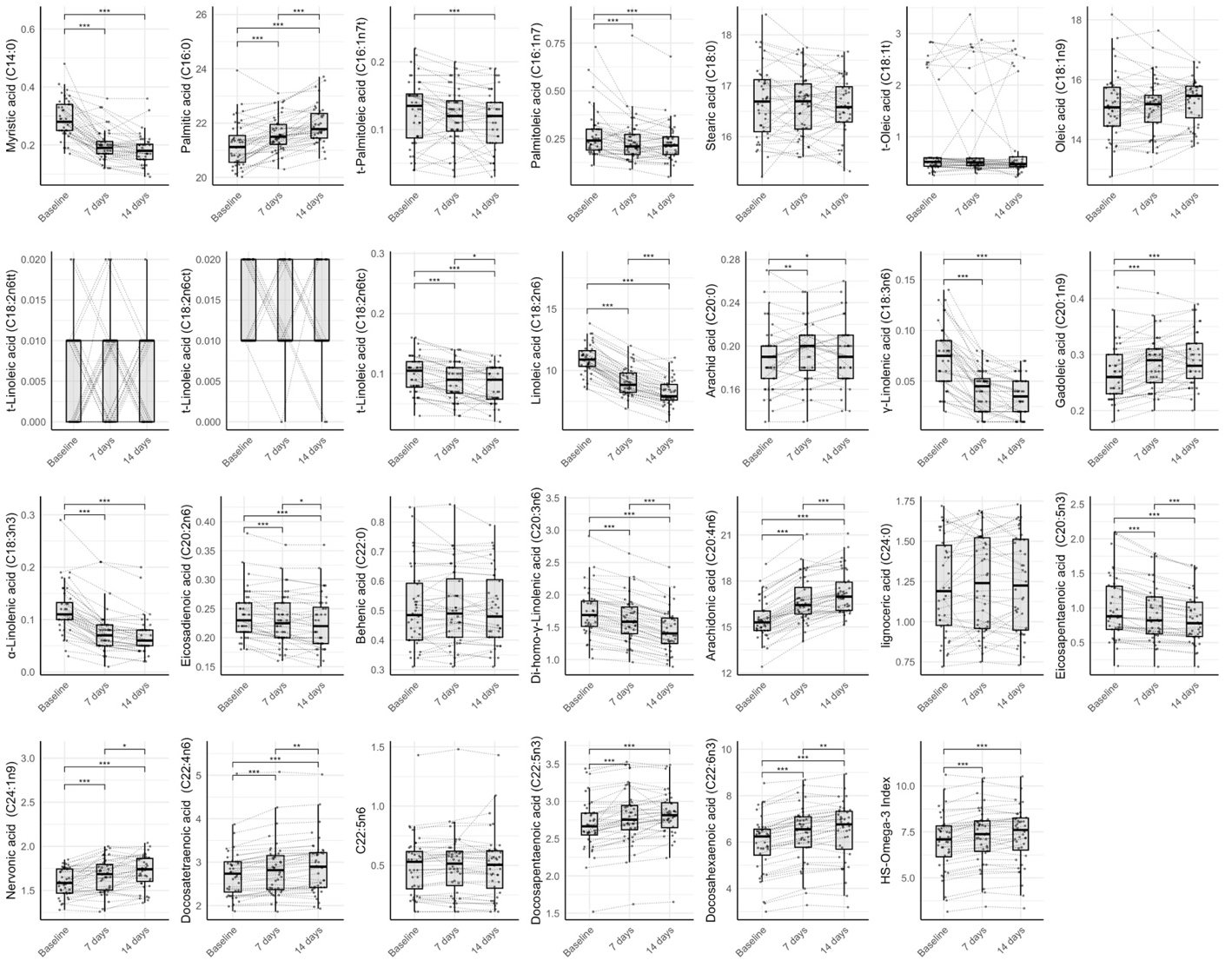
**

Figure S1: Changes in erythrocyte FAs after 7 and 14 fasting days.
